# Supplementary material for: Epizootic ulcerative syndrome causes cutaneous dysbacteriosis in hybrid snakehead (Channa maculata♀ × Channa argus♂)
Source: PeerJ. 2019 Apr 2;7:e6674. doi: 10.7717/peerj.6674 (PMC6450373; doi:10.7717/peerj.6674)

**Supplementary Materials**

Figure S1. Principal co-ordinates analysis (PCoA) profiles based on unweighted (A) and weighted (B) UniFrac distances between samples, and boxplots of the unweighted (C) and weighted (D) UniFrac distances within groups and between groups. Wilcoxon test results of the Unifrac distances within groups and between groups shown in Table S2.


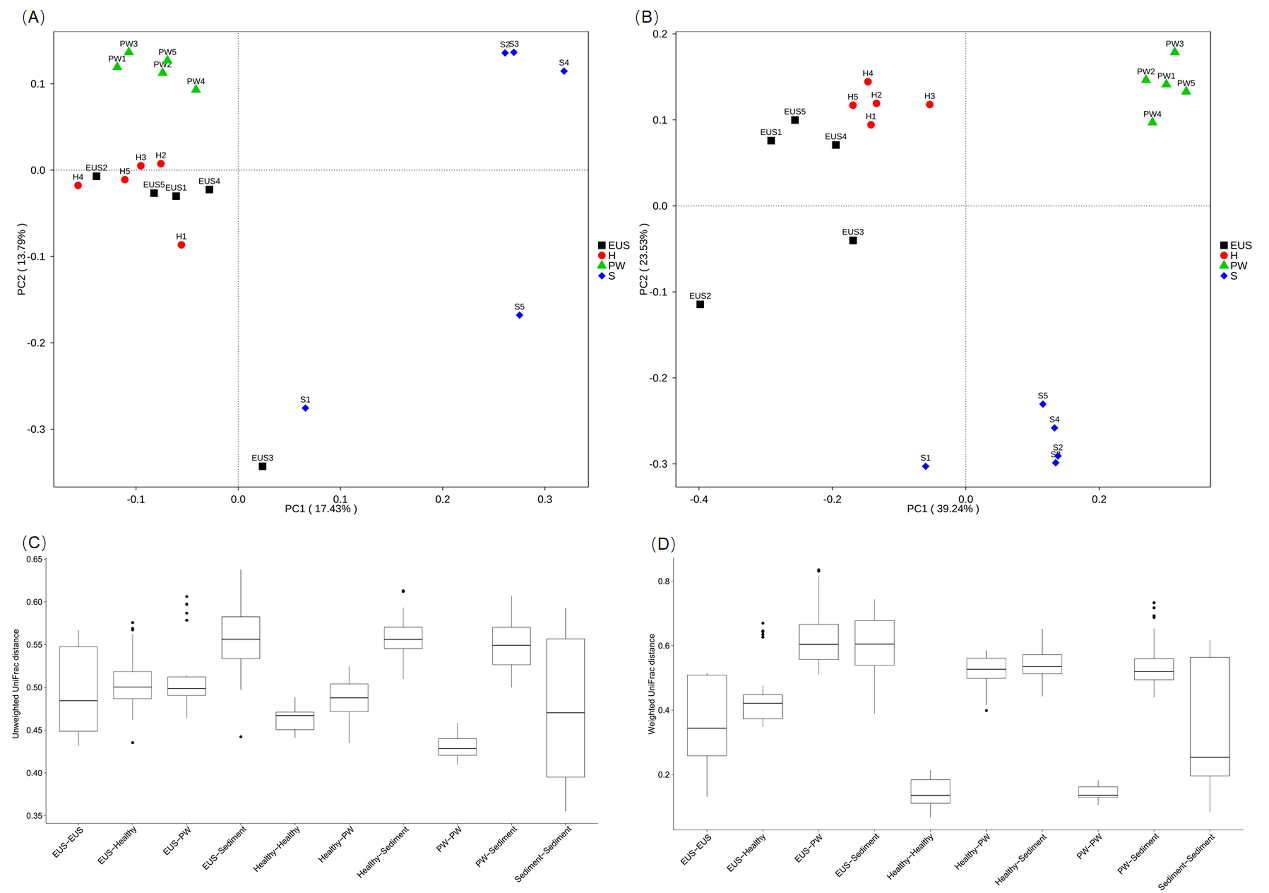

Supplement: Supplemental Information 1 — Wilcoxon test results of the Unifrac distances within groups and between groups shown in Table S2. [file peerj-07-6674-s001.docx]
